# 2-Azido-3,4;6,7-di-*O*-isopropyl­idene-α-d-*glycero*-d-*talo*-heptopyran­ose

**DOI:** 10.1107/S1600536810003995

**Published:** 2010-02-06

**Authors:** Sarah F. Jenkinson, Gabriel M. J. Lenagh-Snow, Ken Izumori, George W. J. Fleet, David J. Watkin, Amber L. Thompson

**Affiliations:** a Department of Organic Chemistry, Chemistry Research Laboratory, University of Oxford, Mansfield Road, Oxford OX1 3TA, England; b Rare Sugar Research Centre, Kagawa University, 2393 Miki-cho, Kita-gun, Kagawa 761-0795, Japan; c Department of Chemical Crystallography, Chemistry Research Laboratory, University of Oxford, Mansfield Road, Oxford OX1 3TA, England

## Abstract

In the title compound, C_13_H_21_N_3_O_6_, the six-membered ring adopts a twist-boat conformation with the azide group in the bowsprit position. The azide group is disordered over two sets of sites in a 0.642 (10):0.358 (10) ratio. The crystal structure consists of O—H⋯O hydrogen-bonded trimer units. The absolute configuration was determined from the use of d-mannose as the starting material.

## Related literature

For Izumoring techniques, see: Izumori (2002 ▶, 2006 ▶); Yoshihara *et al.* (2008 ▶); Gullapalli *et al.* (2010 ▶); Rao *et al.* (2008 ▶, 2009 ▶); Jones *et al.* (2008 ▶); Jenkinson *et al.* (2009 ▶). For the synthesis of homonojirimycins, see: Compain *et al.* (2009 ▶); Asano (2009 ▶); Watson *et al.* (2001 ▶) and for their isolation, see: Ikeda *et al.* (2000 ▶); Asano *et al.* (1998 ▶); Kite *et al.* (1988 ▶). For the synthesis of the azido­heptitol, see: Beacham *et al.* (1991 ▶); Bruce *et al.* (1990 ▶); Myerscough *et al.* (1992 ▶). For the weighting scheme, see: Prince (1982 ▶); Watkin (1994 ▶).
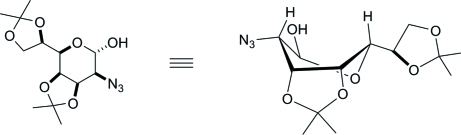


## Experimental

### 

#### Crystal data

C_13_H_21_N_3_O_6_
*M* *_r_* = 315.33
Trigonal, 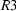

*a* = 16.8793 (2) Å
*c* = 15.1043 (3) Å
*V* = 3726.83 (10) Å^3^
*Z* = 9
Mo *K*α radiation
μ = 0.10 mm^−1^
*T* = 150 K
0.70 × 0.50 × 0.30 mm

#### Data collection

Nonius KappaCCD diffractometer
Absorption correction: multi-scan (*DENZO*/*SCALEPACK*; Otwinowski & Minor, 1997 ▶) *T*_min_ = 0.63, *T*_max_ = 0.97
23870 measured reflections
1889 independent reflections
1770 reflections with *I* > 2σ(*I*)
*R*_int_ = 0.034

#### Refinement

*R*[*F* ^2^ > 2σ(*F* ^2^)] = 0.027
*wR*(*F* ^2^) = 0.070
*S* = 0.87
1889 reflections
227 parameters
43 restraints
H-atom parameters constrained
Δρ_max_ = 0.16 e Å^−3^
Δρ_min_ = −0.16 e Å^−3^

### 

Data collection: *COLLECT* (Nonius, 2001 ▶); cell refinement: *DENZO*/*SCALEPACK* (Otwinowski & Minor, 1997 ▶); data reduction: *DENZO*/*SCALEPACK*; program(s) used to solve structure: *SIR92* (Altomare *et al.*, 1994 ▶); program(s) used to refine structure: *CRYSTALS* (Betteridge *et al.*, 2003 ▶); molecular graphics: *CAMERON* (Watkin *et al.*, 1996 ▶); software used to prepare material for publication: *CRYSTALS*.

## Supplementary Material

Crystal structure: contains datablocks global, I. DOI: 10.1107/S1600536810003995/lh2989sup1.cif

Structure factors: contains datablocks I. DOI: 10.1107/S1600536810003995/lh2989Isup2.hkl

Additional supplementary materials:  crystallographic information; 3D view; checkCIF report

## Figures and Tables

**Table 1:** Hydrogen-bond geometry (Å, °)

*D*—H⋯*A*	*D*—H	H⋯*A*	*D*⋯*A*	*D*—H⋯*A*
O10—H101⋯O6^i^	0.84	1.93	2.761 (3)	171

Symmetry code: (i) 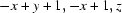.

**Fig. 1.**
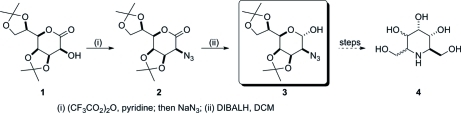
Synthetic Scheme

**Fig. 2.**
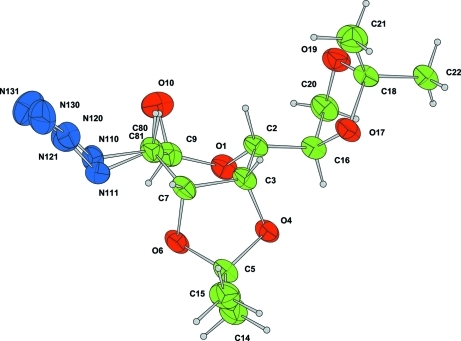
The title compound with displacement ellipsoids drawn at the 50% probability level. H atoms are shown as spheres of arbitary radius.

**Fig. 3.**
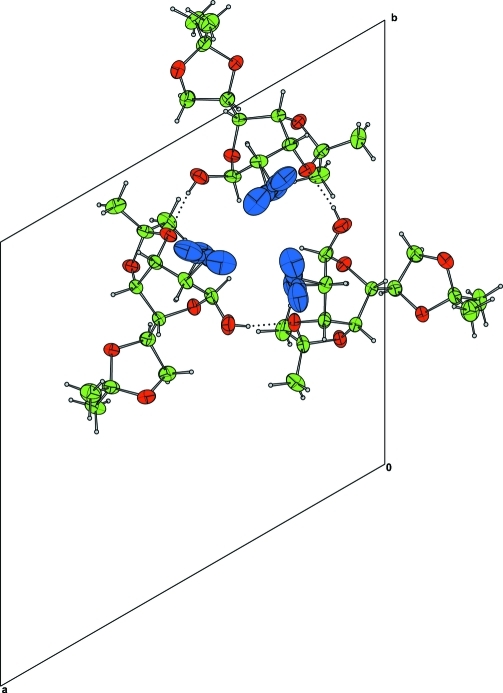
Hydrogen bonded trimer unit. Hydrogen bonds are shown as dotted lines.

**Fig. 4.**
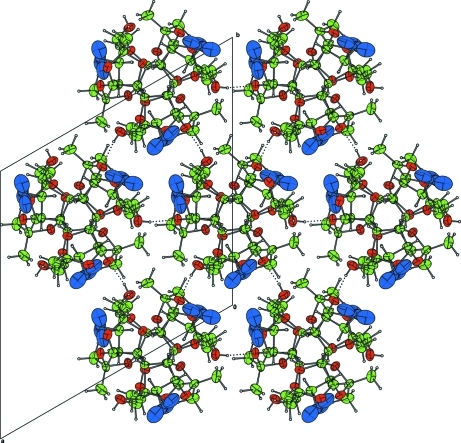
Packing diagram for the title compound projected along the c-axis. Hydrogen bonds are shown by dotted lines.

**Fig. 5.**
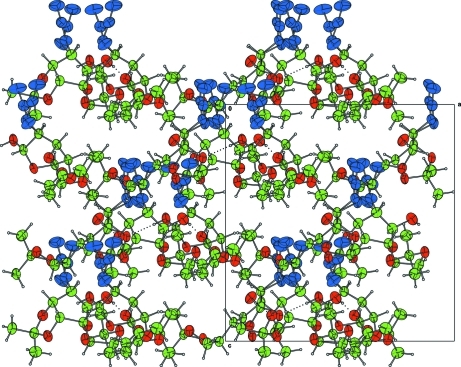
Packing diagram for the title compound projected along the b-axis. Hydrogen bonds are shown by dotted lines.

## supplementary crystallographic information

### Comment

The enzymatic interconversion of monosaccharides has been developed by Izumori (2002, 2006) and has been seen to be generally applicable for the 1-deoxy ketohexoses (Yoshihara *et al.*, 2008, Gullapalli *et al.*, 2010; Rao *et al.*, 2009) and branched sugars (Rao *et al.*, 2008; Jones *et al.*, 2008). The methodology has also been applied to azido heptitols (Jenkinson *et al.*, 2009) and thus to the synthesis of 2,6-dideoxy-2,6-iminoheptitols (homonojirimycins), seven carbon imino sugars (Compain *et al.*, 2009; Asano *et al.*, 2009; Watson *et al.*, 2001) which are a family of glycosidase inhibitors. A number of homonojrimycins have been isolated as natural products from medicinal plants (Ikeda *et al.*, 2000; Asano *et al.*, 1998; Kite *et al.*, 1988).

A Kiliani cyanide reaction on diacetone mannose gave the lactone diacetonide 1 (Beacham *et al.*, 1991; Myerscough *et al.*, 1992). Esterification of 1 (Fig. 1) with triflic anhydride in pyridine followed by reaction with sodium azide in DMF gave the azide 2 with retention of configuration at C2; the stereochemistry of 2 was established by X-ray crystallographic analysis (Bruce *et al.*, 1990). Reduction of the lactone 2 afforded the lactol 3, a key intermediate for the synthesis of four of the possible sixteen iminoheptitols 4 by Izumoring techniques. The reported crystal structure of 3 determines the configuration of both the azide at C2 and the anomeric position.

The X-ray structure shows that the six-membered ring in the title compound adopts a twist boat conformation with the azide in the bowsprit position and the anomeric alcohol group in the less hindered α-position (Fig. 2). There is significant disorder in the structure with the azide occupying two possible sites. The compound exists as repeating hydrogen bonded trimer units (Fig.3, Fig. 4, Fig. 5). The absolute configuration was determined from the use of D-mannose as the starting material. Only classical hydrogen bonding was considered.

### Experimental

The title compound was recrystallised from diethyl ether: m.p. 397-398 K; [α]_D_^25^ +41.3 (*c*, 1.0 in CHCl_3_) {Lit. (Myerscough *et al.*, 1992) m.p. 387-388 K; [α]_D_^20^ +41.0 (*c*, 1.0 in CHCl_3_).

### Refinement

In the absence of significant anomalous scattering, Friedel pairs were merged and the absolute configuration was assigned from the starting material D-mannose. Changes in illuminated volume were kept to a minimum, and were taken into account (Görbitz, 1999) by the multi-scan inter-frame scaling (DENZO/SCALEPACK, Otwinowski & Minor, 1997).

The H atoms were all located in a difference map, but those attached to carbon atoms were repositioned geometrically. The H atoms were initially refined with soft restraints on the bond lengths and angles to regularize their geometry (C—H in the range 0.93–0.98, O—H = 0.82 Å) and *U*_iso_(H) (in the range 1.2–1.5 times *U*_eq_ of the parent atom), after which the positions were refined with riding constraints.

### Crystal data

**Table:**

C_13_H_21_N_3_O_6_	*D*_x_ = 1.264 Mg m^−^^3^
*M**_r_* = 315.33	Mo *K*α radiation, λ = 0.71073 Å
Trigonal, *R*3	Cell parameters from 1885 reflections
Hall symbol: R 3	θ = 5–28°
*a* = 16.8793 (2) Å	µ = 0.10 mm^−^^1^
*c* = 15.1043 (3) Å	*T* = 150 K
*V* = 3726.83 (10) Å^3^	Plate, colourless
*Z* = 9	0.70 × 0.50 × 0.30 mm
*F*(000) = 1512	

### Data collection

**Table:**

Nonius KappaCCD diffractometer	1770 reflections with *I* > 2σ(*I*)
graphite	*R*_int_ = 0.034
ω scans	θ_max_ = 27.5°, θ_min_ = 5.2°
Absorption correction: multi-scan (*DENZO*/*SCALEPACK*; Otwinowski & Minor, 1997)	*h* = −21→21
*T*_min_ = 0.63, *T*_max_ = 0.97	*k* = −21→21
23870 measured reflections	*l* = −19→19
1889 independent reflections	

### Refinement

**Table:**

Refinement on *F*^2^	Primary atom site location: structure-invariant direct methods
Least-squares matrix: full	Hydrogen site location: inferred from neighbouring sites
*R*[*F*^2^ > 2σ(*F*^2^)] = 0.027	H-atom parameters constrained
*wR*(*F*^2^) = 0.070	Method, part 1, Chebychev polynomial, (Watkin, 1994, *P*rince, 1982) [*w*eight] = 1.0/[A_0_*T_0_(x) + A_1_*T_1_(x) ··· + A_n-1_]*T_n-1_(x)] where A_i_ are the Chebychev coefficients listed belo*w* and x = *F* /*F*max Method = Robust Weighting (*P*rince, 1982) W = [*w*eight] * [1-(delta*F*/6*sigma*F*)^2^]^2^ A_i_ are: 35.7 56.9 32.2 12.2 2.39
*S* = 0.87	(Δ/σ)_max_ = 0.0004
1889 reflections	Δρ_max_ = 0.16 e Å^−^^3^
227 parameters	Δρ_min_ = −0.16 e Å^−^^3^
43 restraints	

### Fractional atomic coordinates and isotropic or equivalent isotropic displacement parameters (Å^2^)

**Table:**

	*x*	*y*	*z*	*U*_iso_*/*U*_eq_	Occ. (<1)
O1	0.73085 (8)	0.56052 (8)	0.60278 (11)	0.0388	
C2	0.71163 (11)	0.63258 (11)	0.58727 (14)	0.0334	
C3	0.60997 (11)	0.59051 (10)	0.56713 (13)	0.0348	
O4	0.55748 (8)	0.55027 (8)	0.64559 (12)	0.0420	
C5	0.49780 (12)	0.45418 (11)	0.63308 (14)	0.0398	
O6	0.53523 (9)	0.43128 (8)	0.55852 (11)	0.0414	
C7	0.57940 (12)	0.51014 (10)	0.50233 (14)	0.0370	
C9	0.72913 (13)	0.51340 (12)	0.52439 (14)	0.0407	
O10	0.81499 (10)	0.55590 (10)	0.48357 (13)	0.0511	
C14	0.50399 (17)	0.40362 (15)	0.71240 (16)	0.0540	
C15	0.40195 (14)	0.43489 (16)	0.61352 (17)	0.0544	
C16	0.74277 (12)	0.69311 (12)	0.66864 (14)	0.0376	
O17	0.72041 (8)	0.76403 (8)	0.65905 (12)	0.0420	
C18	0.80336 (12)	0.84914 (11)	0.63848 (14)	0.0408	
O19	0.87138 (8)	0.82461 (9)	0.62410 (12)	0.0457	
C20	0.84636 (13)	0.74546 (14)	0.67731 (16)	0.0468	
C21	0.79042 (17)	0.88864 (16)	0.55377 (18)	0.0588	
C22	0.82716 (16)	0.91340 (13)	0.71627 (16)	0.0519	
H21	0.7470	0.6687	0.5353	0.0391*	
H31	0.5978	0.6378	0.5436	0.0413*	
H71	0.5361	0.5093	0.4580	0.0446*	
H91	0.7123	0.4508	0.5415	0.0490*	
H141	0.4682	0.3379	0.7027	0.0810*	
H142	0.4804	0.4199	0.7637	0.0803*	
H143	0.5683	0.4214	0.7219	0.0805*	
H152	0.3632	0.3698	0.6047	0.0812*	
H153	0.3773	0.4541	0.6630	0.0797*	
H151	0.4011	0.4664	0.5607	0.0801*	
H161	0.7134	0.6569	0.7208	0.0440*	
H201	0.8648	0.7629	0.7392	0.0552*	
H202	0.8739	0.7101	0.6556	0.0567*	
H212	0.8455	0.9466	0.5425	0.0887*	
H213	0.7396	0.9001	0.5601	0.0886*	
H211	0.7793	0.8473	0.5054	0.0875*	
H222	0.8852	0.9682	0.7049	0.0781*	
H223	0.8309	0.8835	0.7700	0.0772*	
H221	0.7802	0.9302	0.7229	0.0777*	
H101	0.8447	0.5336	0.5065	0.0762*	
C80	0.66053 (13)	0.51225 (11)	0.45706 (13)	0.0399	0.642 (10)
N110	0.6365 (5)	0.4337 (5)	0.3965 (5)	0.0459	0.642 (10)
N120	0.6176 (3)	0.4422 (3)	0.3203 (3)	0.0485	0.642 (10)
N130	0.5963 (4)	0.4412 (3)	0.2497 (2)	0.0824	0.642 (10)
C81	0.66053 (13)	0.51225 (11)	0.45706 (13)	0.0399	0.358 (10)
N111	0.6151 (11)	0.4225 (12)	0.4122 (11)	0.0617	0.358 (10)
N121	0.6429 (6)	0.4295 (6)	0.3378 (7)	0.0510	0.358 (10)
N131	0.6661 (7)	0.4252 (6)	0.2652 (4)	0.0936	0.358 (10)
H801	0.6910	0.5680	0.4222	0.0469*	0.642 (10)
H811	0.6898	0.5645	0.4171	0.0473*	0.358 (10)

### Atomic displacement parameters (Å^2^)

**Table:**

	*U*^11^	*U*^22^	*U*^33^	*U*^12^	*U*^13^	*U*^23^
O1	0.0408 (6)	0.0341 (6)	0.0477 (7)	0.0235 (5)	−0.0023 (5)	0.0055 (5)
C2	0.0319 (7)	0.0255 (7)	0.0436 (8)	0.0150 (6)	−0.0009 (6)	0.0042 (6)
C3	0.0331 (8)	0.0254 (7)	0.0461 (9)	0.0149 (6)	−0.0014 (6)	0.0017 (6)
O4	0.0358 (6)	0.0264 (6)	0.0545 (7)	0.0084 (5)	0.0071 (5)	−0.0027 (5)
C5	0.0368 (8)	0.0246 (7)	0.0493 (9)	0.0088 (6)	−0.0017 (7)	−0.0007 (6)
O6	0.0444 (7)	0.0234 (5)	0.0493 (7)	0.0117 (5)	−0.0005 (5)	0.0004 (5)
C7	0.0401 (8)	0.0251 (7)	0.0449 (9)	0.0156 (6)	−0.0042 (7)	0.0014 (6)
C9	0.0454 (9)	0.0324 (8)	0.0518 (10)	0.0251 (7)	0.0036 (8)	0.0077 (7)
O10	0.0522 (8)	0.0543 (8)	0.0632 (8)	0.0390 (7)	0.0123 (7)	0.0181 (7)
C14	0.0670 (13)	0.0407 (10)	0.0523 (12)	0.0254 (10)	−0.0002 (10)	0.0048 (8)
C15	0.0359 (9)	0.0534 (12)	0.0609 (13)	0.0125 (9)	−0.0035 (9)	−0.0009 (9)
C16	0.0341 (8)	0.0311 (7)	0.0438 (9)	0.0133 (7)	−0.0011 (6)	0.0024 (6)
O17	0.0318 (6)	0.0271 (5)	0.0606 (8)	0.0100 (5)	0.0033 (5)	−0.0025 (5)
C18	0.0361 (8)	0.0287 (8)	0.0475 (9)	0.0086 (7)	0.0026 (7)	0.0017 (7)
O19	0.0317 (6)	0.0391 (7)	0.0562 (8)	0.0100 (5)	0.0028 (5)	0.0012 (6)
C20	0.0368 (9)	0.0405 (9)	0.0573 (11)	0.0150 (8)	−0.0072 (8)	−0.0021 (8)
C21	0.0641 (13)	0.0517 (12)	0.0538 (11)	0.0239 (11)	−0.0022 (10)	0.0072 (9)
C22	0.0556 (12)	0.0312 (9)	0.0533 (11)	0.0099 (8)	0.0038 (9)	−0.0034 (8)
C80	0.0489 (10)	0.0299 (8)	0.0448 (9)	0.0226 (7)	−0.0012 (7)	−0.0005 (6)
N110	0.064 (4)	0.033 (3)	0.043 (3)	0.026 (3)	0.004 (3)	−0.0027 (19)
N120	0.049 (2)	0.0377 (17)	0.049 (2)	0.0148 (14)	0.0016 (15)	−0.0079 (14)
N130	0.104 (4)	0.063 (2)	0.052 (2)	0.021 (2)	−0.0091 (19)	−0.0184 (15)
C81	0.0489 (10)	0.0299 (8)	0.0448 (9)	0.0226 (7)	−0.0012 (7)	−0.0005 (6)
N111	0.072 (7)	0.034 (4)	0.060 (6)	0.013 (4)	0.032 (4)	0.001 (3)
N121	0.053 (4)	0.045 (3)	0.052 (4)	0.022 (3)	0.000 (3)	0.002 (3)
N131	0.112 (7)	0.094 (6)	0.050 (4)	0.033 (5)	0.009 (4)	−0.010 (3)

### Geometric parameters (Å, °)

**Table:**

O1—C2	1.4261 (18)	C15—H152	0.966
O1—C9	1.419 (2)	C15—H153	0.985
C2—C3	1.524 (2)	C15—H151	0.963
C2—C16	1.515 (2)	C16—O17	1.431 (2)
C2—H21	0.991	C16—C20	1.520 (2)
C3—O4	1.431 (2)	C16—H161	0.968
C3—C7	1.538 (2)	O17—C18	1.452 (2)
C3—H31	0.986	C18—O19	1.419 (2)
O4—C5	1.4309 (19)	C18—C21	1.508 (3)
C5—O6	1.436 (2)	C18—C22	1.511 (3)
C5—C14	1.505 (3)	O19—C20	1.430 (2)
C5—C15	1.511 (3)	C20—H201	0.983
O6—C7	1.434 (2)	C20—H202	0.978
C7—H71	0.986	C21—H212	0.971
C7—C80	1.515 (3)	C21—H213	0.974
C7—H71	0.986	C21—H211	0.962
C7—C81	1.515 (3)	C22—H222	0.969
C9—O10	1.398 (2)	C22—H223	0.974
C9—H91	0.982	C22—H221	0.971
C9—C80	1.534 (3)	C80—N110	1.491 (8)
C9—O10	1.398 (2)	C80—H801	0.971
C9—H91	0.982	N110—N120	1.221 (8)
C9—C81	1.534 (3)	N120—N130	1.123 (6)
O10—H101	0.838	C81—N111	1.477 (19)
C14—H141	0.974	C81—H811	0.975
C14—H142	0.971	N111—N121	1.201 (16)
C14—H143	0.982	N121—N131	1.179 (13)
			
C2—O1—C9	113.04 (12)	C5—C15—H153	111.3
O1—C2—C3	108.58 (13)	H152—C15—H153	108.8
O1—C2—C16	106.97 (13)	C5—C15—H151	110.7
C3—C2—C16	113.98 (14)	H152—C15—H151	108.9
O1—C2—H21	109.4	H153—C15—H151	108.8
C3—C2—H21	108.7	C2—C16—O17	109.54 (13)
C16—C2—H21	109.2	C2—C16—C20	111.86 (16)
C2—C3—O4	109.97 (14)	O17—C16—C20	103.32 (14)
C2—C3—C7	109.94 (14)	C2—C16—H161	109.8
O4—C3—C7	104.67 (12)	O17—C16—H161	110.2
C2—C3—H31	109.7	C20—C16—H161	111.9
O4—C3—H31	110.6	C16—O17—C18	108.78 (13)
C7—C3—H31	111.8	O17—C18—O19	105.35 (14)
C3—O4—C5	110.27 (13)	O17—C18—C21	109.73 (16)
O4—C5—O6	104.70 (14)	O19—C18—C21	108.44 (17)
O4—C5—C14	109.19 (15)	O17—C18—C22	108.82 (15)
O6—C5—C14	107.89 (15)	O19—C18—C22	111.41 (16)
O4—C5—C15	109.94 (15)	C21—C18—C22	112.81 (17)
O6—C5—C15	110.88 (16)	C18—O19—C20	106.42 (14)
C14—C5—C15	113.82 (17)	C16—C20—O19	102.19 (15)
C5—O6—C7	107.99 (12)	C16—C20—H201	110.8
C3—C7—O6	103.40 (13)	O19—C20—H201	110.7
C3—C7—H71	111.3	C16—C20—H202	112.1
O6—C7—H71	110.5	O19—C20—H202	111.9
C3—C7—C80	111.47 (14)	H201—C20—H202	109.0
O6—C7—C80	109.47 (13)	C18—C21—H212	108.5
H71—C7—C80	110.5	C18—C21—H213	109.9
C3—C7—O6	103.40 (13)	H212—C21—H213	108.2
C3—C7—H71	111.3	C18—C21—H211	110.0
O6—C7—H71	110.5	H212—C21—H211	109.8
C3—C7—C81	111.47 (14)	H213—C21—H211	110.3
O6—C7—C81	109.47 (13)	C18—C22—H222	109.1
H71—C7—C81	110.5	C18—C22—H223	109.8
O1—C9—O10	110.79 (15)	H222—C22—H223	110.3
O1—C9—H91	107.3	C18—C22—H221	109.1
O10—C9—H91	109.7	H222—C22—H221	108.8
O1—C9—C80	111.36 (13)	H223—C22—H221	109.8
O10—C9—C80	107.23 (15)	C9—C80—C7	111.64 (15)
H91—C9—C80	110.5	C9—C80—N110	106.6 (3)
O1—C9—O10	110.79 (15)	C7—C80—N110	114.6 (3)
O1—C9—H91	107.3	C9—C80—H801	108.4
O10—C9—H91	109.7	C7—C80—H801	107.6
O1—C9—C81	111.36 (13)	N110—C80—H801	107.8
O10—C9—C81	107.23 (15)	C80—N110—N120	116.4 (6)
H91—C9—C81	110.5	N110—N120—N130	173.3 (6)
C9—O10—H101	106.7	C9—C81—C7	111.64 (15)
C5—C14—H141	110.3	C9—C81—N111	108.6 (8)
C5—C14—H142	108.7	C7—C81—N111	100.5 (5)
H141—C14—H142	109.5	C9—C81—H811	111.2
C5—C14—H143	108.9	C7—C81—H811	110.2
H141—C14—H143	109.4	N111—C81—H811	114.3
H142—C14—H143	110.1	C81—N111—N121	110.6 (11)
C5—C15—H152	108.4	N111—N121—N131	172.0 (13)

### Hydrogen-bond geometry (Å, °)

**Table:**

*D*—H···*A*	*D*—H	H···*A*	*D*···*A*	*D*—H···*A*
C15—H153···O10^i^	0.99	2.51	3.361 (3)	145
O10—H101···O6^ii^	0.84	1.93	2.761 (3)	171
C80—H801···O4^iii^	0.97	2.36	3.296 (3)	161
C81—H801···O4^iii^	0.97	2.36	3.296 (3)	161
C80—H811···O4^iii^	0.97	2.34	3.296 (3)	166
C81—H811···O4^iii^	0.97	2.34	3.296 (3)	166

Symmetry codes: (i) −*x*+*y*+2/3, −*x*+4/3, *z*+1/3; (ii) −*x*+*y*+1, −*x*+1, *z*; (iii) −*y*+4/3, *x*−*y*+2/3, *z*−1/3.

## References

[bb1] Altomare, A., Cascarano, G., Giacovazzo, C., Guagliardi, A., Burla, M. C., Polidori, G. & Camalli, M. (1994). *J. Appl. Cryst.* **27**, 435.

[bb2] Asano, N. (2009). *Cell. Mol. Life Sci.* **66**, 1479–1492. 10.1007/s00018-008-8522-3 PMC11131492 19132292

[bb3] Asano, N., Nishida, M., Kato, A., Kizu, H., Matsui, K., Shimada, Y., Itoh, T., Baba, M., Watson, A. A., Nash, R. J., Lilley, P. M. D., Watkin, D. J. & Fleet, G. W. J. (1998). *J. Med. Chem.* **41**, 2565–2571. 10.1021/jm970836l 9651160

[bb4] Beacham. A. R., Bruce. I., Choi. S., Doherty. 0., Fairbanks, A. J., Fleet, G. W. J., Skead, B. M., Peach. J. M., Saunders, J. & Watkin, D. J. (1991). *Tetrahedron Asymmetry*, **2**, 883–900.

[bb5] Betteridge, P. W., Carruthers, J. R., Cooper, R. I., Prout, K. & Watkin, D. J. (2003). *J. Appl. Cryst.* **36**, 1487.

[bb6] Bruce, I., Girdhar, A., Haraldsson, M., Peach, J. M., Watkin, D. J. & Fleet, G. W. J. (1990). *Tetrahedron*, **46**, 19–31.

[bb7] Compain, P., Chagnault, V. & Martin, O. R. (2009). *Tetrahedron Asymmetry*, **20**, 672–711.

[bb10] Gullapalli, P., Yoshihara, A., Morimoto, K., Rao, D., Jenkinson, S. F., Wormald, M. R., Fleet, G. W. J. & Izumori, K. (2010). *Tetrahedron Lett.* **51**, 895–898.

[bb11] Ikeda, K., Takahashi, M., Nishida, M., Miyauchi, M., Kizu, H., Kameda, Y., Arisawa, M., Watson, A. A., Nash, R. J., Fleet, G. W. J. & Asano, N. (2000). *Carbohydr. Res.* **323**, 73–80. 10.1016/s0008-6215(99)00246-3 10782288

[bb12] Izumori, K. J. (2002). *Naturwissenschaften*, **89**, 120–124. 10.1007/s00114-002-0297-z 12046631

[bb13] Izumori, K. J. (2006). *Biotechnology*, **124**, 717-722. 10.1016/j.jbiotec.2006.04.016 16716430

[bb14] Jenkinson, S. F., Booth, K. V., Newberry, S., Fleet, G. W. J., Izumori, K., Morimoto, K., Nash, R. J., Jones, L., Watkin, D. J. & Thompson, A. L. (2009). *Acta Cryst.* E**65**, o1755–o1756. 10.1107/S1600536809025045 PMC2977159 21583466

[bb15] Jones, N. A., Rao, D., Yoshihara, A., Gullapalli, P., Morimoto, K., Takata, G., Hunter, S. J., Wormald, M. R., Dwek, R. A., Izumori, K. & Fleet, G. W. J. (2008). *Tetrahedron Asymmetry*, **19**, 1904–1918.

[bb16] Kite, G. C., Fellows, L. E., Fleet, G. W. J., Liu, P. S., Scofield, A. M. & Smith, N. G. (1988). *Tetrahedron Lett.* **29**, 6483–6486.

[bb17] Myerscough, P. M., Fairbanks, A. J., Jones, A. H., Bruce, I., Choi, S. S., Fleet, G. W. J., Al-Daher, S. S., Cenci di Bello, I. & Winchester, B. (1992). *Tetrahedron*, **48**, 10177–10194.

[bb18] Nonius (2001). *COLLECT* Nonius BV, Delft, The Netherlands.

[bb19] Otwinowski, Z. & Minor, W. (1997). *Methods in Enzymology*, Vol. 276, *Macromolecular Crystallography*, Part A, edited by C. W. Carter Jr & R. M. Sweet, pp. 307–326. New York: Academic Press.

[bb20] Prince, E. (1982). *Mathematical Techniques in Crystallography and Materials Science.* New York: Springer-Verlag.

[bb21] Rao, D., Best, D., Yoshihara, A., Gullapalli, P., Morimoto, K., Wormald, M. R., Wilson, F. X., Izumori, K. & Fleet, G. W. J. (2009). *Tetrahedron Lett.* **50**, 3559–3563.

[bb22] Rao, D., Yoshihara, A., Gullapalli, P., Morimoto, K., Takata, G., da Cruz, F. P., Jenkinson, S. F., Wormald, M. R., Dwek, R. A., Fleet, G. W. J. & Izumori, K. (2008). *Tetrahedron Lett.* **49**, 3316–3121.

[bb23] Watkin, D. (1994). *Acta Cryst.* A**50**, 411–437.

[bb24] Watkin, D. J., Prout, C. K. & Pearce, L. J. (1996). *CAMERON* Chemical Crystallography Laboratory, Oxford, England.

[bb25] Watson, A. A., Fleet, G. W. J., Asano, N., Molyneux, R. J. & Nash, R. J. (2001). *Phytochemistry*, **56**, 265–295. 10.1016/s0031-9422(00)00451-9 11243453

[bb26] Yoshihara, A., Haraguchi, S., Gullapalli, P., Rao, D., Morimoto, K., Takata, G., Jones, N., Jenkinson, S. F., Wormald, M. R., Dwek, R. A., Fleet, G. W. J. & Izumori, K. (2008). *Tetrahedron Asymmetry*, **19**, 739–745.

